# A new mutually reinforcing network node and link ranking algorithm

**DOI:** 10.1038/srep15141

**Published:** 2015-10-23

**Authors:** Zhenghua Wang, Leonardo Dueñas-Osorio, Jamie E. Padgett

**Affiliations:** 1 Department of Civil and Environmental Engineering, Rice University, Houston, TX 77005, USA; 2 Department of Civil and Environmental Engineering, Rice University, Houston, TX 77005, USA; 3 Department of Civil and Environmental Engineering, Rice University, Houston, TX 77005, USA

## Abstract

This study proposes a novel Normalized Wide network Ranking algorithm (NWRank) that has the advantage of ranking nodes and links of a network simultaneously. This algorithm combines the mutual reinforcement feature of Hypertext Induced Topic Selection (HITS) and the weight normalization feature of PageRank. Relative weights are assigned to links based on the degree of the adjacent neighbors and the Betweenness Centrality instead of assigning the same weight to every link as assumed in PageRank. Numerical experiment results show that NWRank performs consistently better than HITS, PageRank, eigenvector centrality, and edge betweenness from the perspective of network connectivity and approximate network flow, which is also supported by comparisons with the expensive *N*-1 benchmark removal criteria based on network efficiency. Furthermore, it can avoid some problems, such as the Tightly Knit Community effect, which exists in HITS. NWRank provides a new inexpensive way to rank nodes and links of a network, which has practical applications, particularly to prioritize resource allocation for upgrade of hierarchical and distributed networks, as well as to support decision making in the design of networks, where node and link importance depend on a balance of local and global integrity.

Ranking individual elements within networks, including both nodes and links, allows for the identification of important subsets of elements for resource prioritization, such as finding the most authoritative webpages related to a search topic in the internet, discovering the most influential people in a social network, evaluating the most cited scientific papers in a citation network, or identifying the most vulnerable components in infrastructure systems (e.g., transportation networks, power grids, and water systems). Quantification of the criticality of network components helps decision makers inform their management strategies. For example, infrastructure designers can set mandatory safety targets or reliability levels for components (e.g., highways and bridges) in a transportation network, despite resources being typically limited. Therefore, computationally efficient measures that can identify or screen out the importance of network nodes *and* links are required, as standard methods are too computationally demanding.

The ranking of network nodes or links addresses the question “Can important nodes or links in a network be meaningfully identified while keeping input data and computational resources low?” To answer this question, several closed-form ranking measures have been proposed to capture the particular features of networks, mainly based on their topology^1^,^2^,^3^,^4^,^5^. For example, one of the simplest node importance measures in a network is the degree of a node *k*_*i*_, denoting the number of links connected to it. Traditionally, the adjacency matrix ***A*** is used to describe the connectivity patterns of nodes and links of a network. The adjacency matrix has also been used as input for advanced eigenvalue and eigenvector analyses, which continue to gain adoption in practice^6^. Spectral network component rankings are sophisticated extensions of fundamental ranking methodologies, such as node degree centrality *k*_*i*_^7^,^8^. One of the most successful developments in spectral ranking of network components has been the PageRank algorithm^3^, which forms the basis of Google webpage rankings, where websites are nodes and hyperlinks are the connections among them. PageRank considers the hyperlink weight normalization and web surfing principles based on random walk models^9^. Another popular ranking algorithm is the Hypertext Induced Topic Selection (HITS) algorithm developed by Kleinberg^4^. The HITS algorithm defines two types of nodes in a network, *hubs* and *authorities*, and computes the ranking score of them in a mutually reinforcing way. However, both PageRank and HITS are limited to ranking nodes of a network and they do not account for the importance of links. Very few efficient algorithms for the ranking of links exist in the literature.

Ranking studies for webpage networks and citation networks have mainly focused on the ranking of nodes because link ranking in such networks is not of practical interest. This is in sharp contrast to certain types of networks, such as transportation networks, whose links are at least as important as nodes, as well as supply chain networks, whose links are important to find the movement of a product or service to the end customer. However, relatively limited research has been performed so far to investigate the ranking of the links of a network. Betweenness centrality of an edge has been used as an approximate indicator of the link’s critical role in the passage of network flow^10^,^11^. Several other studies rank the links of networks based on algorithms whose premise is that the most vital links in a network are those whose one-by-one removal result in the greatest change of a system-level performance metric, such as decrease of the efficiency, connectivity loss, or increase of the travel time^12^,^13^. The *N*-1 criterion in power systems is a practical example of this approach^14^. Even though the measure of performance of the network may vary, the basic idea for prioritizing the components of the network based on the removal of its components is essentially the same across available studies^15^,^16^. However, these link-based measures usually depend on the calculation of network performance (e.g., connectivity reliability and network flow) after the removal of the links, which is time-consuming and depends on the choice of the performance measures. For example, for the whole transportation network of the United States, there are tens of thousands of nodes. Hence, the traditional removal strategy (e.g., based on *N*-1 criteria) is too time-consuming for practical applications.

Therefore, new computationally efficient tools, particularly based on the spectral analysis of networks for joint node-link ranking are needed. This link-aware ranking strategy must remain computationally feasible even for large systems, which is a persistent challenge in infrastructure engineering today^16^. This paper develops new ranking indices by building upon the promising spectral properties of networks. First, this study explores the formulation of a ranking index called Wide Rank (WRank) that can rank the nodes *and* links of a network simultaneously. Building upon this WRank formalism, a Normalized WRank algorithm (NWRank) is proposed in this manuscript. This NWRank index combines the idea of mutual reinforcement and weight normalization into a unified framework. Then, the WRank and NWRank approaches are applied to a broad set of synthesized network models and compared with the PageRank, HITS, eigenvector centrality, and edge betweenness algorithms, as well as the benchmark *N*-1 criterion based on a network efficiency metric, which is widely used in the network science and engineering fields to study the performance of various kinds of networks.

## Results

### WRank algorithm as a building block

Let *G* (***N***, ***M***) represent a network consisting of a node set ***N*** with *n* nodes and a link set ***M*** with *m* links. In addition, it is assumed that no node is directly connected to itself and that multiple links do not exist between nodes. In the WRank algorithm proposed here, instead of focusing solely on the ranking of nodes as emphasized in previous studies, each node *i* ∈ ***N***receives a ranking score *x*_*i*_ and each link *a* ∈ ***M*** also receives a ranking score *y*_*a*_. The premise is that an important node should be pointed to by many critical links (this defines the *L* operation below), and a critical link should point to important nodes (this defines the *Q* operation below). The mutually reinforcing relationship of nodes and links through the noted operators can be represented in general as follows,


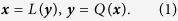


where vectors ***x*** = (*x*_1_, *x*_2_, *…*, *x*_*n*_)^*T*^ and ***y*** = (*y*_1_, *y*_2_, *…*, *y*_*m*_)^*T*^ contain the ranking score of each node and link, respectively. The mutual reinforcement operations *L* and *Q* can be expressed through the following matrix representations:


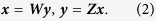


where ***W*** is a *n* × *m* matrix and ***Z*** is a *m* × *n* matrix whose generic entries are given by


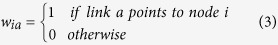



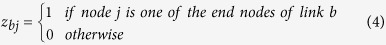


where *i*, *j*, *k*, … ∈ ***N*** are nodes and *a*, *b*, *c*, … ∈ ***M*** are links. It should be noted that ***W*** is the transposed matrix of ***Z***, and vice versa. Therefore, once one of them is obtained, the other one can be easily obtained. The final ranking scores of each node and link can be obtained through an iterative updating process indexed by *t*. Similar to the HITS algorithm^4^, the ranking vectors ***x*** and ***y*** need to be normalized so that their sum is equal to 1 before moving to the next iteration *t* + 1. If ***x***(*t*) and ***y***(*t*) are used to denote ranking scores at the *t*^*th*^ iteration, the iterative processes to reach the final solutions are:


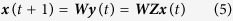



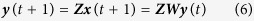


The final solutions ***x***, ***y*** converge to the principal eigenvectors of ***WZ*** and ***ZW*** or to the linear combination of the principal eigenvectors if more than one principal eigenvector exists, similar to HITS-inspired algorithms. It should be noted that although ***WZ*** is similar to the adjacency matrix, the diagonal entries are different. The diagonal entries of the adjacency matrix are zeros but the diagonal entries of ***WZ*** are non-zeros. The diagonal entries of the ***WZ*** matrix are the in-degrees of the corresponding nodes, which makes the WRank algorithm suitable for directed networks automatically. Hence, ***WZ*** provides node-to-node connectivity as well as in-degree in the diagonal, while ***ZW*** provides link-to-link connectivity as well as link multiplicity in the diagonal.

### Proposed NWRank algorithm

**Figure 1:**
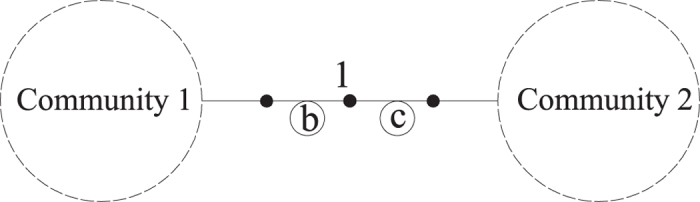
A low degree node that is important for network flow or its approximations, but missed in PageRank and HITS rankings.

Although the WRank algorithms can work well for most types of networks, two problems could arise for certain networks with prestigious nodes or where network flow (or its approximation) is considered in the ranking of nodes and links. First, if a node with a high ranking score is pointed to by many links, then all of those links also obtain high ranking scores. However, this is not necessarily appropriate. The score received by each link from a prestigious node should be diluted by being shared with others. To solve similar problems, PageRank weights each of the out-going hyperlinks from a webpage, *p*_*i*_, by 1/*k*_*i*_, where *k*_*i*_ is the degree of node *p*_*i*_; thus, every link has the same total out-going weight equal to 1/*k*_*i*_. Even though the PageRank algorithm is used successfully in Google, it does not work in the WRank algorithms. It turns out that every link ends up with exactly the same ranking score if each link has the same out-going weight 1/*k*_*i*_. In fact, both the PageRank and HITS algorithms treat all links equally when distributing ranking scores. However, in practice some links are more important than others. A second challenge is that none of the closed-form network measures, except for Betweenness Centrality approximately, capture the role of network flow in the ranking of nodes or links. Consider the situation depicted in Fig. 1 where node 1 lies on a path between two communities. Although node 1 has a low degree, it may have considerable impact on the flow between other nodes within the network. In addition, links *b* and *c* also have significant influence on the network flow. However, both PageRank and HITS algorithms do not consider the network flow in the ranking of nodes and only nodes that are well-connected with others obtain a high ranking score. Therefore, node 1 has a very low ranking value if PageRank or HITS algorithms are used to rank the nodes.

To address the two problems stated above, this study expands the WRank formulation into a weight-normalized algorithm referred to as NWRank. Instead of dividing the ranking score of the nodes evenly among each incident link, each link obtains a weight proportional to its neighboring nodes' degree and the Betweenness Centrality (BC) of the link. The link will obtain more weight if the adjacent neighbor nodes have a high degree, the BC of the link is high, or both. In the new method, the ***Z*** matrix is replaced by the following ***H*** matrix:


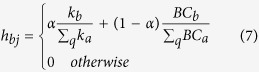


where *h*_*bj*_ is the score distribution from node *j* ∈ ***N*** to link *b* ∈ ***M***, *q* is the number of links that connect to node *j*; *k*_*b*_ and *k*_*a*_ represent the degree of the end nodes opposite to *j* of links *b* and *a* = *1*, *…*, *q*, respectively. Also, *BC*_*b*_ and *BC*_*a*_ are the Betweenness Centrality levels of links *b* and *a* = *1*, *…*, *q*, respectively. The variable *α* is the weight coefficient. In the case study, equal weights (i.e. *α* = 0.5) are chosen between the degree and *BC* as a starting point (unbiased point), acknowledging that connectivity and network flow contribute equally; in practice, methods based on utility theory or other user-based elicitation strategies can be adopted to establish weights^17^. For now, sensitivity studies are conducted to analyze the influence of the weighting coefficients on the ranking results (presented in the following section). It should be noted that the ***W*** matrix can also be modified to include the node betweenness and node degree similar to the ***H*** matrix. However, the final ranking score of nodes and links did not change much based on the case study networks introduced below. Therefore, in this study the ***W*** matrix is kept unaltered. In sum, for the NWRank algorithm, the iterative processes to reach the final solutions is:


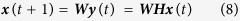



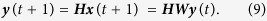


Note that the NWRank algorithm combines the most important mutual reinforcement feature of HITS and the most important hyperlink weight normalization feature of PageRank as well as the consideration of approximate network flow into a unified framework to provide an efficient joint node-link ranking. The mutual reinforcement here refers to the node-link relationship instead of the node-node relationship in HITS algorithms, which is one of the novelties of the NWRank algorithm. In addition, the weight normalization approach proposed here assigns more weight to important links based on both the topology of the network and an approximate form of the network flow through it, making the method applicable to different network problems where both nodes and links are critical for system performance, and where a trade-off between local and global integrity is at work, owing to connectivity and flow considerations, respectively.

### Application to diverse networks

To evaluate the WRank algorithm and, in particular, the NWRank algorithm, they are used along with PageRank, HITS, eigenvector centrality, and edge betweenness to study several diverse idealized networks. In order to further evaluate the NWRank algorithm, all the above algorithms are also compared with traditional *N-1* removal-based strategies. Different metrics can be used to quantify the network performance in the removal strategy depending on the types of networks. For example, travel time may be used in transportation networks and cost may be used in supply chain networks. As conceived, NWRank is a general ranking algorithm that can be used to rank the nodes or links of generic networks. Therefore, a general metric (i.e. network efficiency *E*^18^ as shown in Equation 10) is used for assessment and comparisons here, where *N* is the number of nodes in the network, and *d*_*ij*_ is the shortest distance between pairs of nodes in a network. Network Efficiency *E*^18^ has been used to describe the performance of various kinds of networks^19^,^20^,^21^,^22^,^23^,^24^, as well as the performance of general complex networks^25^,^26^,^27^,^28^. Therefore, network efficiency is a popular metric to describe the network performance in the network science and engineering applications.


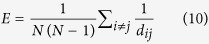


Real world complex networks are usually the result of integrating several idealized yet topologically diverse networks of different sizes and configurations. For example, the transportation network of the United States consists of states’ transportation networks. The states’ transportation networks are made up by the cities’ transportation networks, and so on. The rule also applies to other networks, such as the internet, social networks, telecommunication networks, water systems and power grids. Therefore, investigating the configuration of the building blocks (i.e. typical small networks with some homogeneous features) found in large networks is informative^29^. Yazdani *et al*.^30^ divided a subset of typical small diverse networks into two main categories that capture different network configurations: (1) hierarchical network models, where the networks are organized (and possibly operated) in a hierarchical fashion with some of the nodes easily identifiable as the master or upstream nodes while most other nodes are flow distribution junctions and downstream nodes; and (2) distributed network models, where the networks have a relatively uniform topology and only a few hubs or single failure-points are present. This study uses these small yet topologically diverse ensembles of networks, along with a few other ideal topologies (e.g., grids, and scale-free networks), to test the performance of the WRank and NWRank algorithms.

### Computational experiments with hierarchical networks

**Figure 2:**
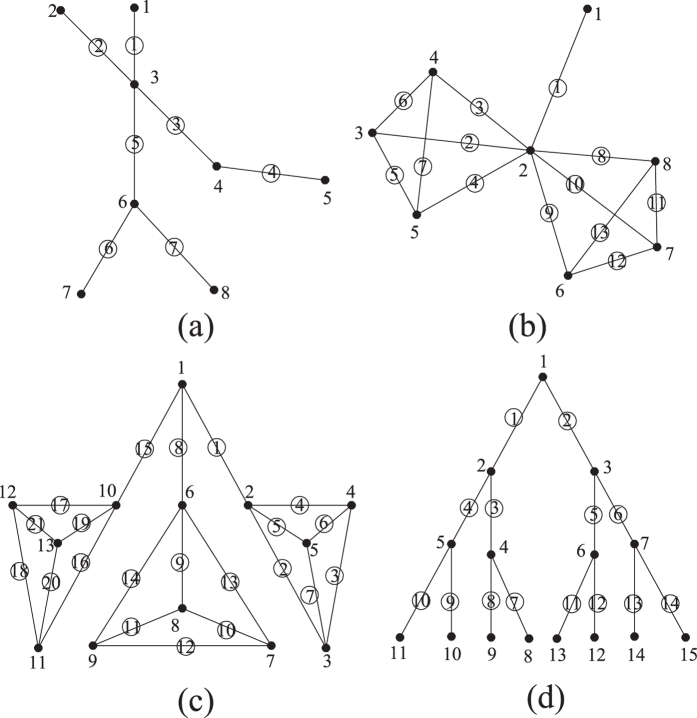
Topology of select hierarchical networks: (a) 8-node simple transportation network (T8); (b) 8-node “bat” network (B8) (c) 13-node three-cluster network (C13) which is symmetric under each link emanating from node 1, and (d) symmetric 15-node tree network (HT15).

The configuration of the hierarchical networks is likely seen within many spatially organized technological and urban infrastructure systems composed of transmission (global) and distribution (local) subsystems. Four hierarchical networks are selected as case studies and their idealized topologies are shown in Fig. 2. The basic information about these four networks is listed in Table 1.

Tables 2 and 3 list the node and link ranking results of the four hierarchical networks. Nodes and links that have the same score are not separated by horizontal lines. For simple hierarchical networks (e.g., T8 and B8), the node ranking of the NWRank algorithm is consistent with the WRank, HITS, and eigenvector centrality algorithms. Therefore, the weight normalization of the link has minor influence on the ranking of the nodes for simple hierarchical networks. Also, it can be seen that the node ranking for WRank and NWRank relative to the HITS algorithm across the other hierarchical networks (e.g., C13 and HT15) is almost the same due to their common mutual reinforcement feature, although some differences are observed as these networks start benefiting from a joint node and link ranking perspective. For instance, in C13 which has clear clusters, it is more beneficial to maintain the node linking such clusters (node 1), whereas in HT15 with no such clusters, it is more beneficial to have some nodes enabling network subsets to remain connected (nodes 2 and 3)—as captured by NWRank and not by HITS. The NWRank also performs better than the naïve WRank, HITS, and eigenvector centrality algorithms based on the results from an *N-1* removal strategy as well as a perspective of network integrity where local and global connectivity trade-offs are at work. For example, Tables 2 and 3 show that for node ranking, NWRank obtains the same ranking results with the *N-1* removal strategy for the four hierarchical networks. This is in contrast with other node rankings. For example, while the node ranking of B8 for the PageRank algorithm is the same as the other three algorithms, for most other networks it produces different node ranking results which do not reflect the role of network flow in the ranking of nodes and links. Specifically, the PageRank algorithm ranks node 1 as the least important node of C13, which is at odds from the perspective of network connectivity and network flow. This is because for the PageRank algorithm, the ranking of nodes is highly correlated with the ranking by the in-degree of nodes^33^,^34^,^35^. Node 1 has relatively small in-degree in C13, and it appears at the bottom of the ranking list.

Regarding the ranking of links, NWRank is the only algorithm that obtains the same ranking results with the *N-1* removal strategy for most of the hierarchical networks (i.e. T8, C13, and HT15), with a difference in B8 where NWRank favors edges to maintain clusters (edges 2–4 and 8–10) to keep most of the network integrated at the expense of leaving some nodes connected with lower-ranked links (depending on edge 1 in this case, which is favored by the *N*-1 and betweenness strategies). In addition, except for HT15, WRank and NWRank provide the same link ranking results, where the difference stems from the trade-off mechanism of NWRank to handle local and global connectivity perspectives, where topology dominates the local aspect and flow the global one. Also, the NWRank algorithm obtains the same link ranking results with edge betweenness algorithms for C13 and HT15. Across the four networks, it can be seen that the NWRank algorithm aligns either with the WRank algorithm or the edge betweeness algorithm because it combines the features of both of them, while revealing expected edge rankings each time (i.e., favoring integrity within clusters over entire network connectivity when network leafs of weakly connected subsets are present). This is captured in the importance of links 1, 8, and 15 and node 1 of C13, or links 1 and 2 and nodes 2 and 3 of HT15. The superiority of the NWRank algorithm for the ranking of nodes and links is further confirmed below with other complex networks, such as distributed networks, which tend not to have obvious prestigious nodes.

### Computational experiments with distributed networks

**Figure 3:**
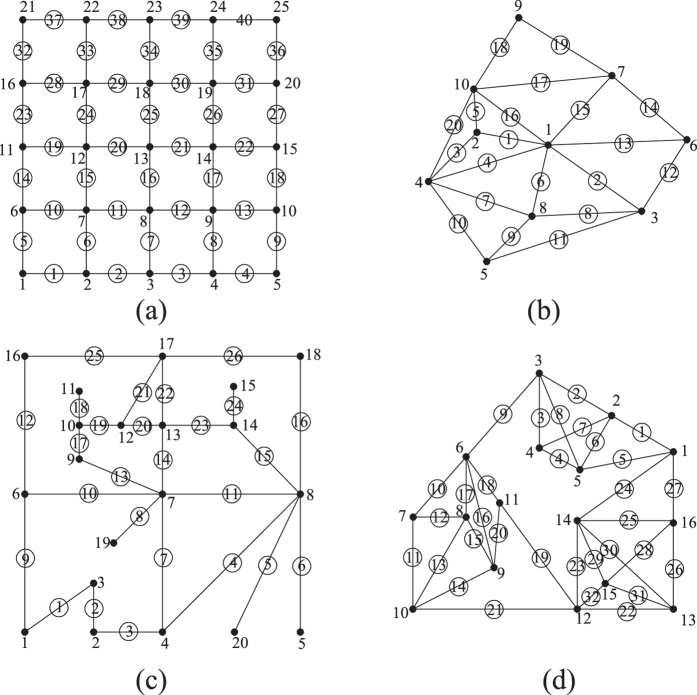
Topology of select distributed networks: (**a**) 25-node grid network (Grid 25); (**b**) 10-node Delaunay Triangulation (DT10); (**c**) 20-node Scale-Free distributed network (SF20); (**d**) 16-node uniform distributed network (UD16).

While it is relatively easy to identify the master nodes or links for the hierarchical networks, it is usually more difficult to identify the authoritative components for distributed networks, such as sensor networks and certain types of transportation and commodity distribution networks. Different from the hierarchical networks, distributed operation is usually self-organized, which allows for a greater allocation of redundancy and operation of alternative supply paths^30^. Four distributed networks are chosen in this study and their topologies are shown in Fig. 3. The basic information of these networks is listed in Table 4. To facilitate ranking comparisons across strategies, the ranking agreement between all the ranking algorithms with the *N-1* removal strategy is quantified by cosine similarity^37^ for distributed networks.

While Tables 5 and 6 list the node and link ranking results of the four distributed networks, Table 7 and Table 8 synthesize comparisons between all the ranking algorithms and the *N-1* removal-based approach. It can be seen that for node ranking, NWRank is the only ranking algorithm that obtains high cosine similarities with the traditional *N*-1 removal strategy for all the four distributed networks. The cosine similarity values are at least 0.856, indicating good agreement. While other ranking algorithms can obtain similar ranking results with the removal strategy for certain distributed networks, they are not consistent across all networks, especially for the UD16 network as explained later. As with the hierarchical networks, for certain distributed networks the node ranking of WRank and HITS algorithms is very similar (e.g. identical for Grid25, and similar for DT10, SF10, and UD16). In addition, the eigenvector centrality and HITS obtain the same node ranking results for the four distributed networks. Also, based on Table 7, PageRank still does not perform well for distributed networks. Because NWRank combines the mutual reinforcement feature of HITS and WRank as well as the weight normalization feature of PageRank, the node ranking produced by NWRank is in between the rankings produced by WRank, HITS, eigenvector centrality, and PageRank as shown by the SF20 and UD16 networks.

For the link ranking, the NWRank algorithm is in between the WRank and edge betweenness algorithms because it combines the features of both of them. Based on Table 8, the NWRank algorithm still performs better than the WRank algorithm and the edge betweenness algorithms for most of the distributed networks, especially for UD16 network. UD16 is a good example that illustrates the potential problems of the naïve WRank and HITS algorithms. It is easy to identify that three communities exist in the UD16 network. For the link ranking of UD16, the top 11 links produced by the WRank algorithm all belong to the same community while the top 11 links produced by the NWRank algorithm distribute among all the three communities, especially the links that connect the communities, which reflects the importance of approximate network flow. The same is true for the ranking of nodes. The WRank and HITS algorithms lead to very similar rankings and all the top ranking nodes belong to the same communities. However, NWRank (and PageRank) finds the top ranking nodes from all of the three communities. This problem for the ranking of nodes in HITS-inspired algorithms, including the naïve WRank approach, was originally called the Tightly Knit Community (TKC) effect by Lempel and Moran^38^. A tightly knit community is a small but highly interconnected set of nodes and the TKC effect occurs when such community scores high in link-analyzing algorithms^38^. This effect hampers the ability of the mutual reinforcement approach to identify meaningful authorities^35^. NWRank is less vulnerable to the TKC effect, and can find meaningful authorities where the mutual reinforcement approach (WRank and HITS algorithms) fails to do so.

The difference between rankings produced by different algorithms reflects the different features of each ranking algorithm. The most important feature of HITS and WRank algorithms is the mutual reinforcement of nodes or nodes and links, while the salient feature of PageRank is the weight normalization of the link. Edge betweenness approximately considers the role of network flow in the ranking. However, the NWRank algorithm combines the features of both mutual reinforcement from HITS and weight normalization from PageRank. In addition, NWRank also approximately considers the network flow in the simultaneous ranking of nodes and links, which is desirable in certain networks like infrastructure systems, where links carry commodities. Such link-centric information has not been traditionally featured by other ranking approaches. In addition, NWRank can obtain similar ranking results relative to the expensive *N*-1 removal based strategy, which is used as a benchmark for ranking comparison herein. However, NWRank is much more efficient than the traditional removal strategy and produces satisfactory results that emphasize integrity balancing local and global perspectives. NWRank tends to favor network integrity in a practical sense (favoring a large percentage of the network to be connected even if a small percentage is disconnected, which agrees with engineering design principles; whereas other rankings tend to favor elements that keep the entire network connected not admitting a suboptimal state). For instance, the link rankings for DT10 and SF20 show NWRank favoring edges that keep network integrity in a large percentage, whereas other rankings, including the *N*-1 approach based on efficiency favors links connecting to terminal nodes. Similar but more subtle trends are measured for the Grid25 and UG16 link rankings. NWRank prefers large partitions over large networks, which is desirable because NWRank considers network connectivity and network flow in the ranking of nodes and links.

To highlight the computational efficiency of NWRank, a relatively large transportation network of South Carolina with 1,486 nodes and 2,321 links is selected to test the computational requirements of NWRank. It takes 1,551 seconds to calculate the ranking results if a common *N*-1 removal strategy is used, while it only takes 9.1 seconds if the NWrank algorithm is used. For very large networks, such as the whole transportation network of the United States and the internet a traditional removal strategy (e.g., *N*-1 criterion) may become too time consuming for practical implementations. In addition to an absolute time perspective of computational efficiency, the algorithm for *N*-1 ranking mainly depends on shortest path finding algorithms and the calculation of efficiency for every element removed one at a time, making the overall worst case time complexity proportional to *O*(*N*^4^)^39^. In contrast, NWRank’s main operation is a matrix multiplication of arrays on the order of *N* requiring *O*(*N*^2.4^) to *O*(*N*^3^) time (depending on the implementation), and the evaluation of betweenness, which scales as *O*(*N*^3^)^40^ for an overall computational complexity of *O*(*N*^3^).

In addition, although not shown here, NWRank has been used in other real networks by the authors. The authors have used NWRank algorithm to rank the nodes and links of the transportation network of Charleston, South Carolina^31^ with 185 nodes and 279 links. Results show that NWRank can give reasonable link ranking results of the transportation network and the cosine similarity values relative to the N-1 criterion are 0.87 and 0.85 for the node and link ranking, respectively. Further details on the ranking of the transportation networks can be found in Wang 2014^31^. In addition, the authors have used NWRank algorithm to rank the nodes and links of a power grid network with 88 nodes and 98 links, and compared it with the *N*-1 criteria. Results show that the cosine similarity values are 0.98 and 0.95 for the node and link ranking, respectively. Beyond the hierarchical and distributed networks presented here, the outcomes of the case study real transportation network and power grid network suggest that NWRank can be used to efficiently and effectively rank the nodes and links of typical large networks, such as large infrastructure systems.

### Sensitivity of the weight coefficients on the ranking results

In order to evaluate the influence of the weight coefficients in Equation (7) on the ranking of nodes and links, another four *α* and 1-*α* pairs [i.e. (1, 0); (0.75, 0.25); (0.25, 0.75); (0, 1)] in addition to (0.5, 0.5) are used in the NWRank algorithm to rank the network nodes and links. Results show that if *α* is large, network connectivity plays a great role which means that nodes with high degree and associated links rank high. If 1-*α* is large, approximate network flow has more effect on the ranking which means that the nodes and links with high betweenness values rank high. In addition, the difference between the ranking score of links decreases with the increase in the weight of *α* versus 1-*α*. Given the results of the sensitivity study, the selection of the weight coefficients should be conducted according to the decision makers’ preference. If network connectivity is more important, relatively larger *α* values [e.g., (0.75, 0.25)] can be used. If network flow is more important, relatively smaller *α* values [e.g., (0.25, 0.75)] can be used. Otherwise, equal weight (i.e. *α* = 0.5) can be chosen as an unbiased point.

## Discussion

This study proposes a new mutually reinforcing network ranking algorithm (NWRank) which combines the mutual reinforcement feature of HITS and the weight normalization feature of PageRank and also considers the role of network flow approximately. This new algorithm is different in that it can rank the nodes and links of a network simultaneously, which is important for certain types of networks, particularly engineering infrastructure systems whose links are as critical as nodes. Numerical experiment results show that NWRank can obtain similar node ranking results relative to the HITS and WRank algorithms for networks without the TKC effect or prestigious nodes. For networks with TKC effects or prestigious nodes, NWRank can avoid the problem that exists in the HITS and WRank algorithms. For the node ranking, NWRank is somewhere in-between the rankings produced by HITS, WRank, eigenvector centrality, and PageRank, and performs better than the four algorithms because it combines the advantages of both HITS and PageRank algorithms while avoiding the TKC effect. For the link ranking, NWRank is better than WRank and edge betweenness based on the comparison with an *N*-1 expensive removal strategy, and it can capture features of both network connectivity and approximate network flow as shown with the networks in this study. NWRank enhances upon the current practical or state-of-the-art topological methods on network component ranking in terms of scope, computational complexity, and applicability. NWRank provides a new way to rank nodes and links of a network efficiently, which has practical applications in many types of networks and queries. For instance, to find cost-effective paths in supply chain networks, or to prioritize resource allocation for upgrade of infrastructure systems, such as transportation networks, water systems, and power grids among others, all with the aim to support decision making and design, particularly when balancing local connectivity and global flow. Also, if detailed information of the networks is available (e.g., link and node capacities, node supply and demand levels, and physics-based governing flow models), future work can compare approximate topological ranking strategies, as NWRank, with *N*-1 removal strategies measuring performance as capacitated networks that cannot violate physical constraints.

## Additional Information

**How to cite this article**: Wang, Z. *et al*. A new mutually reinforcing network node and link ranking algorithm. *Sci. Rep*. **5**, 15141; doi: 10.1038/srep15141 (2015).

## Figures and Tables

**Table 1:** Basic information about selected hierarchical networks.

Name	*n*	*m*	Reference
Transportation network (T8)	8	7	31
Bat network (B8)	8	13	32
Three-cluster network (C13)	13	21	30
Hierarchical tree network (HT15)	15	14	30

**Table 2:**
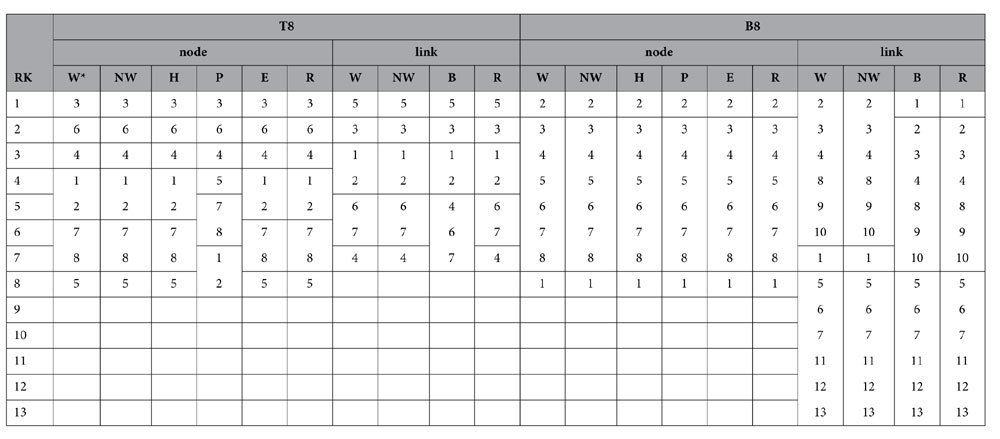
Node and link ranking results for the T8 and B8 hierarchical networks.

*W = WRank; NW = NWRank; P = PageRank; H = HITS; E = eigenvector centrality; B = edge betweenness; R = *N-1* removal strategy.

**Table 3:**
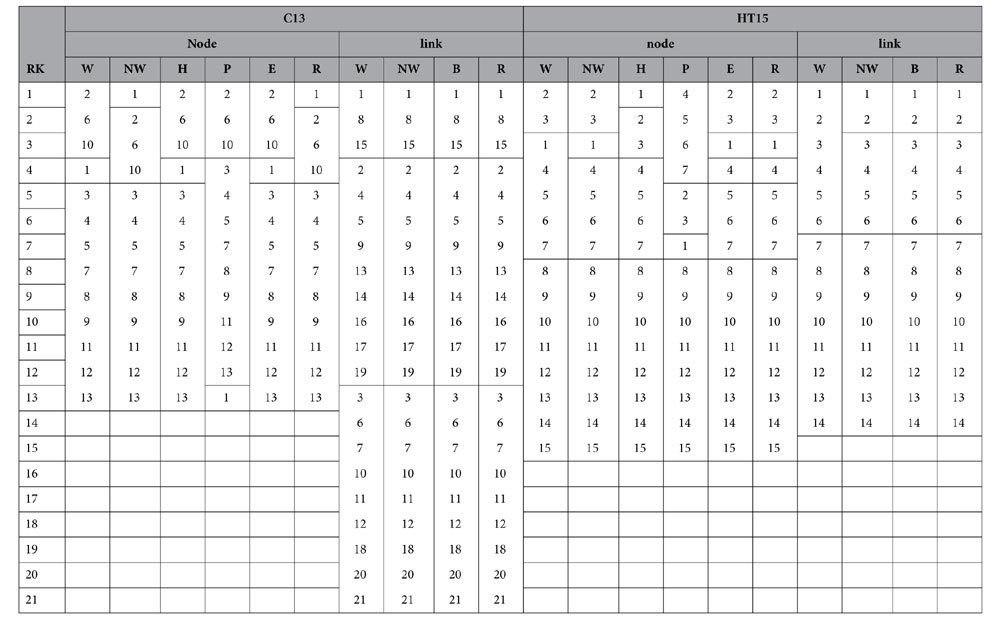
Node and link ranking results for the C13 and HT15 hierarchical networks.

**Table 4:** Basic information of the selected distributed networks.

Name	*n*	*m*	Reference
Grid network (Grid25)	25	40	*
Delaunay Triangulation network (DT10)	10	20	36
Scale-free distributed network (SF20)	20	26	*
Uniform distributed network (UD16)	16	32	29

*developed by the author and his collaborators.

**Table 5:**
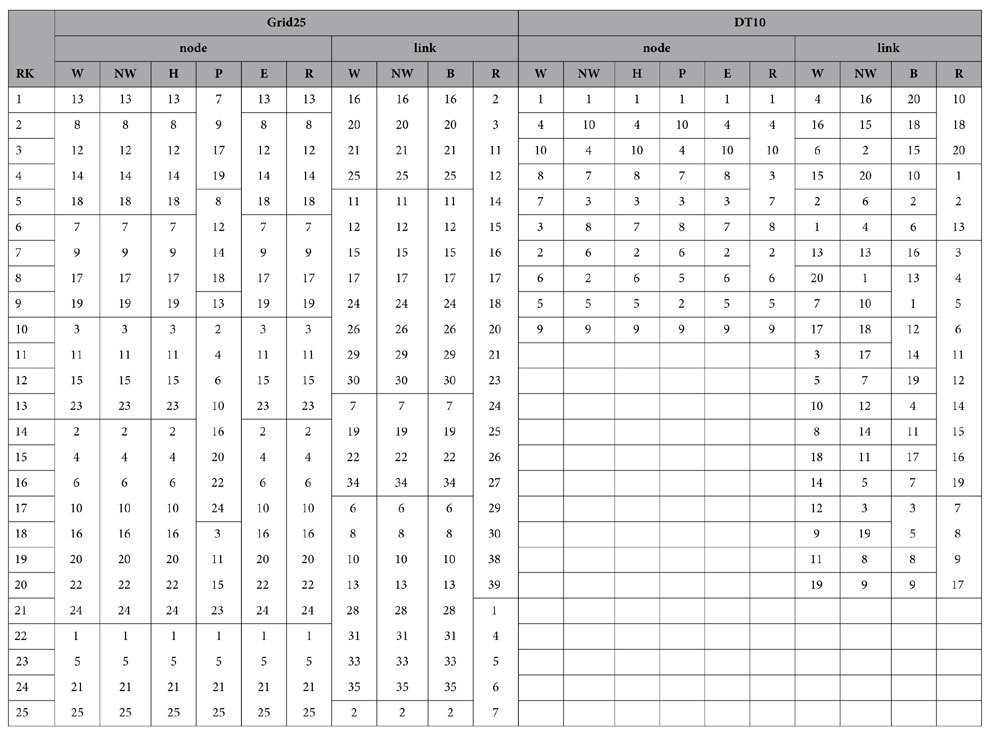
Node and link ranking results of the Grid25 and DT10 distributed networks.

**Table 6:**
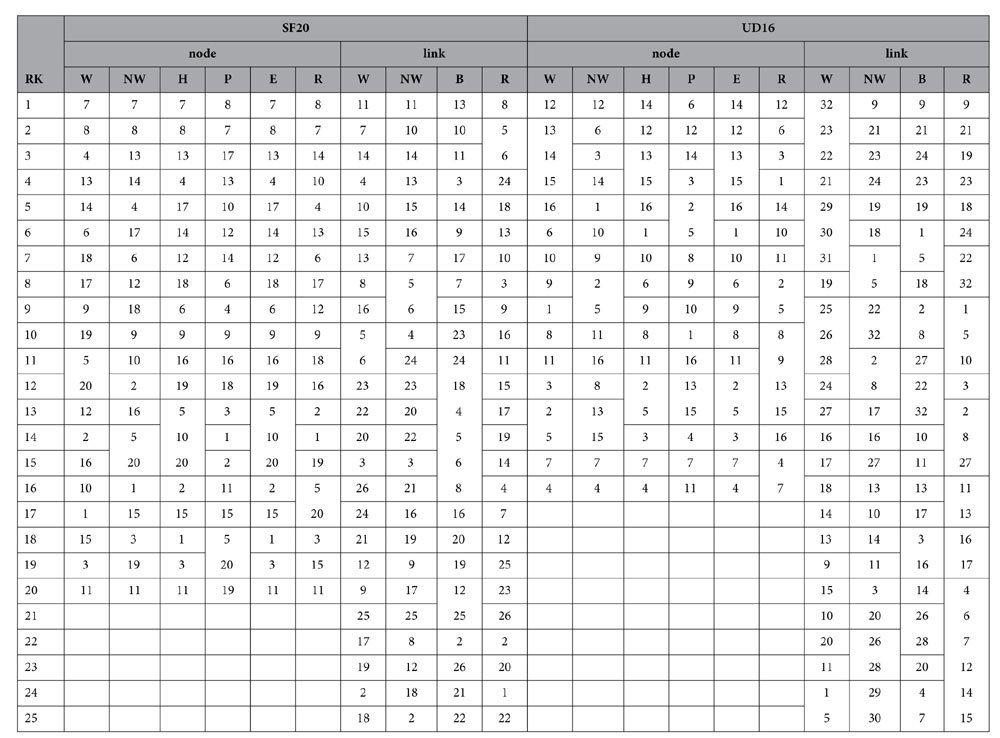
Node and link ranking results of the SF20 and UD16 distributed networks.

**Table 7:** Comparison of the node ranking between the ranking algorithms and *N*-1 removal strategy based on cosine similarity with the boldface used to demonstrate the most similar ranking.

Network	Grid25	DT10	SF20	UD16
NWRank	**1.0**	0.867	0.891	**0.856**
WRank	**1.0**	0.833	0.742	0.717
PageRank	0.839	0.831	0.868	0.753
HITS	**1.0**	**0.946**	**0.901**	0.717
Eigen vector	**1.0**	**0.946**	**0.901**	0.717

**Table 8:** Comparison of the link ranking between the ranking algorithms and *N*-1 removal strategy based on cosine similarity with the boldface used to demonstrate the most similar ranking.

Network	Grid25	DT10	SF20	UD16
NWRank	**0.953**	**0.957**	0.932	**0.903**
WRank	**0.953**	0.934	0.800	0.667
Edge betweenness	**0.953**	0.951	**0.945**	0.814
